# Buprenorphine Dispensed by Pharmacies and Administered in Emergency Departments in Urban and Rural Areas — United States, 2019–2025

**DOI:** 10.15585/mmwr.mm7533a2

**Published:** 2026-08-27

**Authors:** Gery P. Guy, Yijie Chen, Kun Zhang, Nisha Nataraj, Macarena C. Garcia, Deborah Dowell, Grant Baldwin

**Affiliations:** ^1^National Center for Injury Prevention and Control, Division of Overdose Prevention, CDC; ^2^Public Health Infrastructure Center, Office of Rural Health, CDC.

**Summary**

**What is already known about this topic?**

In 2024, a total of 54,045 persons died from opioid-involved drug overdoses in the United States. Buprenorphine, a Food and Drug Administration–approved medication for opioid use disorder, reduces opioid use and related deaths but remains underused.

**What is added by this report?**

Buprenorphine dispensing by pharmacies increased during 2019–2021, particularly in rural counties, before declining through 2025, although rates remained higher in rural than in urban counties. Emergency department (ED) adoption and administration of buprenorphine for opioid use disorder or nonfatal opioid overdoses also increased but remained consistently lower in rural counties.

**What are the implications for public health practice?**

Expanding access to buprenorphine in urban and rural counties, including increasing the number of prescriptions, improving availability in pharmacies, supporting ED-based treatment, and sustaining telehealth flexibilities, could improve treatment access and reduce overdose risk.

## Abstract

Drug overdose deaths remain a major public health concern in the United States. Although buprenorphine is approved by the Food and Drug Administration to treat opioid use disorder and reduces overdose risk and deaths, the medication remains underused in the United States, and many persons who could benefit from treatment lack access. This report describes 2019–2025 trends in buprenorphine dispensing by pharmacies, treatment initiation among persons newly receiving buprenorphine, treatment retention, pharmacy availability, and emergency department (ED) administration by urban-rural county classification using IQVIA data on buprenorphine dispensed by pharmacies and the Premier Healthcare Database. Dispensing rates increased from 2019 to 2021, before declining thereafter, and remained consistently higher in rural counties than in urban counties. The number of patients per prescriber and prescriptions per prescriber declined in both urban and rural counties. Initiation and retention were higher in rural counties; initiation declined in urban counties, and retention declined over time in both urban and rural counties. Pharmacy availability of buprenorphine increased in both urban and rural counties. Adoption and administration of ED buprenorphine increased in both urban and rural counties but remained lower in rural counties. These findings highlight differences between urban and rural areas in buprenorphine access and treatment, with more reliance on pharmacies dispensing buprenorphine in rural counties and higher ED-based administration in urban counties. Opportunities to improve access include use of a low threshold for prescribing buprenorphine, strengthening pharmacy availability of buprenorphine, and (particularly in rural areas) supporting ED-based treatment and sustaining telehealth flexibilities.

## Introduction

Drug overdose deaths remain a major public health concern in the United States. In 2024, opioid-involved deaths accounted for 54,045 (68.1%) overdose deaths (Drug Overdose Deaths | National Center for Health Statistics | CDC). Also in 2024, an estimated 4.8 million persons had a diagnosed opioid use disorder (OUD) (2024 National Survey on Drug Use and Health | Substance Abuse and Mental Health Services Administration). Medications for OUD reduce opioid use, overdose risk, and deaths (*1*); however, only approximately one in four persons with OUD receives medication for OUD (*2*).

Buprenorphine, the most prescribed of the three Food and Drug Administration (FDA)–approved medications for OUD, can be prescribed in clinical settings and dispensed through retail pharmacies. Emergency department (ED) administration of buprenorphine after nonfatal overdose or OUD-related visits can also facilitate linkage to treatment. However, pharmacy dispensing rates and ED administration have changed little in recent years (*3*,*4*), and buprenorphine remains underused (*2*).

Geographic variation in access might contribute to underuse, reflecting differences in prescriber and pharmacy availability and health system capacity. This report describes trends in buprenorphine dispensing, initiation, retention, and ED administration during 2019–2025 by urban-rural county classification using national pharmacy and hospital data.

## Methods

### Data Sources

**Pharmacy dispensing.** Data on pharmacy-dispensed buprenorphine during 2019–2025 were obtained from IQVIA,* a U.S. health information technology and clinical research company that includes approximately 94% of U.S. retail pharmacy prescriptions. Measures included dispensing rates calculated per 1,000 persons using annual U.S. Census Bureau population estimates, days’ supply per prescription, average daily dose per prescription, prescribers (health care providers who prescribed at least one buprenorphine prescription in a year) per 1,000 persons, patients per prescriber, prescriptions per prescriber, percentage of prescriptions from the top 10% of prescribers (prescribers whose annual buprenorphine prescriptions were >90th percentile among all buprenorphine prescribers during that year), percentage of prescriptions from emergency medicine settings (i.e., emergency medicine specialists and clinicians practicing in EDs), and pharmacy availability of buprenorphine (defined as a pharmacy dispensing at least one buprenorphine prescription during every month a pharmacy was in operation in a given year).^†^ Monthly initiations per 100,000 persons (receipt of a buprenorphine prescription by a patient who had not received a prescription during the preceding 180 days) and 180-day retention (receipt of a continuous 180-day supply, which could be achieved through one or more prescriptions and refills, without a gap of >7 days) were evaluated. Initiations during July–December 2025 were excluded to ensure 180 days of follow-up data for all patients included in the analysis.

**ED administration.** ED-administered buprenorphine data were obtained from the Premier Healthcare Database, an all-payer database including approximately 8 million inpatient admissions and 86 million outpatient visits annually from approximately 1,400 hospitals. Measures included annual rates of buprenorphine administration (administrations per 1,000 ED visits and administrations per 1,000 OUD or nonfatal opioid overdose visits) and ED adoption (administration of buprenorphine to at least one person each month during which an OUD or overdose visit occurred). An OUD visit was defined as a visit with at least one *International Classification of Diseases, Tenth Revision, Clinical Modification* *(ICD-10-CM)* diagnosis code in the F11 category. A nonfatal opioid overdose visit was defined as a visit with at least one *ICD-10-CM* diagnosis code for opium (T40.0), heroin (T40.1), natural and semisynthetic opioids (T40.2), methadone (T40.3), synthetic opioids other than methadone (T40.4), or other and unspecified narcotics (T40.6).

### Analysis

Measures were stratified by 2020 U.S. Census Bureau urban-rural county classification using prescriber location for dispensing data and ED location for ED-administered buprenorphine.^§^ Trends were calculated using Joinpoint (version 5.0; National Cancer Institute) regression and reported as average annual percent change (AAPC) or average monthly percent change (AMPC), and segment-specific trends were reported as annual percent change (APC) or monthly percent change (MPC). Analyses were conducted using Stata (version 17.0; StataCorp), Spark SQL (version 3.5.0; The Apache Software Foundation) and Joinpoint (version 5.4.0; National Cancer Institute). This activity was reviewed by CDC, deemed not research, and conducted consistent with applicable federal law and CDC policy.^¶^

## Results

### Retail Pharmacy Dispensing

**Dispensing rates in rural and urban counties.** Buprenorphine pharmacy dispensing rates increased from 2019 to 2021, and rate increases were more pronounced in rural counties (APC = 7.0) than in urban counties (APC = 0.7). During 2021–2025, pharmacy dispensing rates declined in both rural and urban counties; rates remained higher in rural counties despite decreasing more rapidly in rural counties (Table). During 2019–2025, the average prescription supply increased from 17.6 to 22.6 days in urban counties and from 17.4 to 22.2 days in rural counties; the percentage of prescriptions for ≥15 days increased from 45.1% to 65.2% in urban counties and from 30.7% to 53.7% in rural counties. Modest increases in average daily dose per prescription were noted**.**

**TABLE:** Percentage change in buprenorphine dispensed by retail pharmacies and administered in emergency departments in urban and rural areas,* by year — United States, 2019–2025

Characteristic	Year							AAPC	APC^†^ (95% CI)
	2019	2020	2021	2022	2023	2024	2025		
**Dispensed by retail pharmacies**									
Dispensing rate per 1,000 persons									
Urban	46.4	46.9	46.9	46.3	44.9	44.1	42.9	–1.3 (–1.4 to –1.2)	2019–2021: 0.7 (0.5 to 0.9) 2021–2025: –2.3 (–2.4 to –2.2)
Rural	51.8	56.5	58.3	58.3	56.4	53.4	51.1	–0.2 (–0.9 to 0.5)	2019–2021: 7.0 (3.3 to 9.7) 2021–2025: –3.6 (–5.1 to –2.7)
Days’ supply per prescription									
Urban									
Mean	17.6	19.2	19.6	20.6	21.0	21.9	22.6	3.9 (3.4 to 4.2)	2019–2021: 5.3 (4.1 to 6. 6) 2021–2025: 3.2 (1.7 to 3.6)
Percentage									
≤7 days	36.7	30.9	28.3	26.0	23.3	21.7	19.9	—	—
8–14 days	18.2	18.5	18.0	17.4	16.9	16.0	14.9	—	—
≥15 days	45.1	50.6	53.7	56.7	59.8	62.3	65.2	—	—
Rural									
Mean	17.4	18.5	19.3	20.3	20.8	21.6	22.2	4.17 (4.1 to 4.2)	2019–2021: 5.8 (5.4 to 6.0) 2021–2025: 3.4 (3.2 to 3.5)
Percentage									
≤7 days	47.1	43.0	40.6	37.5	33.0	29.9	27.6	—	—
8–14 days	22.2	22.6	21.1	20.9	20.3	19.3	18.7	—	—
≥15 days	30.7	34.4	38.3	41.6	46.6	50.8	53.7	—	—
Average daily dose per prescription, mg									
Urban	14.6	14.8	14.8	15.0	15.1	15.2	15.5	0.8 (0.7 to 1.0)	2019–2022: 0.5 (0.1 to 0.8) 2022–2025: 1.2 (0.9 to 1.6)
Rural	14.8	14.9	14.9	15.1	15.2	15.4	15.6	0.9 (0.8 to 1.0)	2019–2023: 0.7 (0.5 to 0.8) 2023–2025: 1.3 (1.0 to 1.5)
No. of prescribers per 1,000 persons									
Urban	0.1	0.1	0.2	0.2	0.3	0.3	0.4	19.0 (17.1 to 21.1)	2019–2021: 10.8 (4.7 to 17.6) 2021–2025: 23.3 (20.8 to 29.8)
Rural	0.1	0.1	0.1	0.1	0.2	0.2	0.2	20.7 (18.7 to 22.7)	2019–2022: 17.1 (9.9 to 22.3) 2022–2025: 24.5 (19.4 to 31.9)
Patients per prescriber^§^									
Urban	48.7	46.8	44.2	41.2	30.9	27.3	25.7	–11.0 (–12.9 to 9.6)	2019–2021: –3.9 (–9.9 to 1.9) 2021–2025: –14.4 (–19.6 to –12.6)
Rural	60.1	57.4	58.9	58.3	46.0	39.0	36.3	–8.7 (–11.4 to –5.9)	2019–2022: –1.9 (–6.2 to 10.1) 2022–2025: –15.1 (–24.7 to –11.3)
Prescriptions per prescriber^§^									
Urban	312.8	295.5	260.1	228.5	156.0	131.1	116.0	–16.4 (–18.4 to –14.9)	2019–2021: –8.3 (–15.0 to –2.0) 2021–2025: –20.2 (–25.6 to –18.3)
Rural	469.2	460.9	430.7	391.6	282.1	228.3	195.8	–14.0 (–15.4 to 13.0)	2019–2021: –1.7 (–8.3 to 2.7) 2021–2025: –19.6 (–22.5 to –18.1)
Percentage of buprenorphine prescriptions from top 10% of prescribers^¶^									
Urban	71.1	71.1	72.9	75.0	82.0	84.3	84.8	3.7 (2.3 to 5.1)	—
Rural	65.6	65.5	65.8	67.5	76.3	79.3	81.0	4.0 (3.3 to 4.9)	2019–2021: –0.1 (–2.6 to 2.9) 2021–2025: 6.3 (5.2 to 8.3)
Percentage of buprenorphine prescriptions from emergency medicine settings**									
Urban	3.5	3.1	3.0	3.0	2.9	3.0	3.0	–2.3 (–2.9 to –1.6)	2019–2021: –6.9 (–8.6 to –4.3) 2021–2025: 0.1 (–0.9 to 1.7)
Rural	5.2	3.9	3.2	3.0	2.7	2.5	2.2	–12.6 (–13.5 to –11.2)	2019–2021: –19.8 (–22.7 to –15.0) 2021–2025: –8.8 (–10.5 to –5.0)
**Administered in EDs**									
ED visits with buprenorphine administered per 1,000 ED visits									
Urban	0.3	0.5	0.6	0.7	0.7	0.9	1.0	18.4 (16.0 to 20.5)	2019–2021: 30.34 (21.5 to 38.6) 2021–2025: 12.88 (7.1 to 15.1)
Rural	0.4	0.5	0.5	0.5	0.5	0.6	0.7	6.9 (4.8 to 8.3)	2019–2023: 2.2 (–2.3 to 4.1) 2023–2025: 16.8 (9.2 to 22.8)
Buprenorphine administration rate per 1,000 ED visits for OUD or nonfatal opioid overdose^††^									
Urban	33.9	38.9	51.1	62.8	74.8	97.0	111.1	23.3 (22.5 to 24.0)	—
Rural	25.2	39.0	37.3	47.1	62.9	87.1	103.3	25.1 (20.9 to 28.7)	2019–2022: 18.1 (4.6 to 28.0) 2022–2025: 32.4 (22.1 to 46.3)

**Abbreviations**: AAPC = average annual percent change; APC = annual percent change; ED = emergency department; *ICD-10-CM* = *International Classification of Diseases, Tenth Revision, Clinical Modification*; OUD = opioid use disorder.

**Source:** IQVIA Xponent, IQVIA Longitudinal Prescription Database, IQVIA Prescriber Level Xponent, and Premier Healthcare Database, 2019–2025.

* The U.S. Census Bureau defines an urban area as a territory in which core census groups or blocks have a population density of at least 1,000 persons per square mile, and surrounding census blocks have an overall density of at least 500 persons per square mile. Rural areas are considered territory outside the definition of urban.

^†^ APCs represent segment-specific trends in which any Joinpoint is identified by the regression model, and AAPCs represent the estimated trend over the entire study period.

^§^ Prescribers are health care providers who prescribed at least one buprenorphine during a year.

^¶^ Top 10% is defined as prescribers whose annual buprenorphine prescriptions were above the 90th percentile of all buprenorphine prescribers in that year.

** Percentage of all buprenorphine prescriptions prescribed in emergency medicine settings (i.e., emergency medicine specialists and clinicians practicing in EDs).

^††^ An OUD visit was defined as any visit with at least one *ICD-10-CM* diagnosis code in the F11 category. A nonfatal opioid overdose visit was defined as any visit with at least one *ICD-10-CM* diagnosis code for opium (T40.0), heroin (T40.1), natural and semisynthetic opioids (T40.2), methadone (T40.3), synthetic opioids other than methadone (T40.4), or other and unspecified narcotics (T40.6).

**Buprenorphine prescribers.** From 2019 to 2025, the number of prescribers per 1,000 persons increased in both urban counties (from 0.1 to 0.4) and rural counties (from 0.1 to 0.2) at the same time that the number of patients per prescriber and the number of prescriptions per prescriber decreased, with patients per prescriber decreasing from 48.7 to 25.7 in urban counties and from 60.1 to 36.3 in rural counties and prescriptions per prescriber decreasing from 312.8 to 116.0 in urban counties and from 469.2 to 195.8 in rural counties. Among buprenorphine prescriptions dispensed from retail pharmacies, the percentage prescribed by the top 10% of prescribers increased from 71.1% to 84.8% in urban counties and from 65.6% to 81.0% in rural counties, whereas the percentage prescribed in emergency medicine settings decreased from 3.5% to 3.0% in urban counties and from 5.2% to 2.2% in rural counties.

**FIGURE 1:**
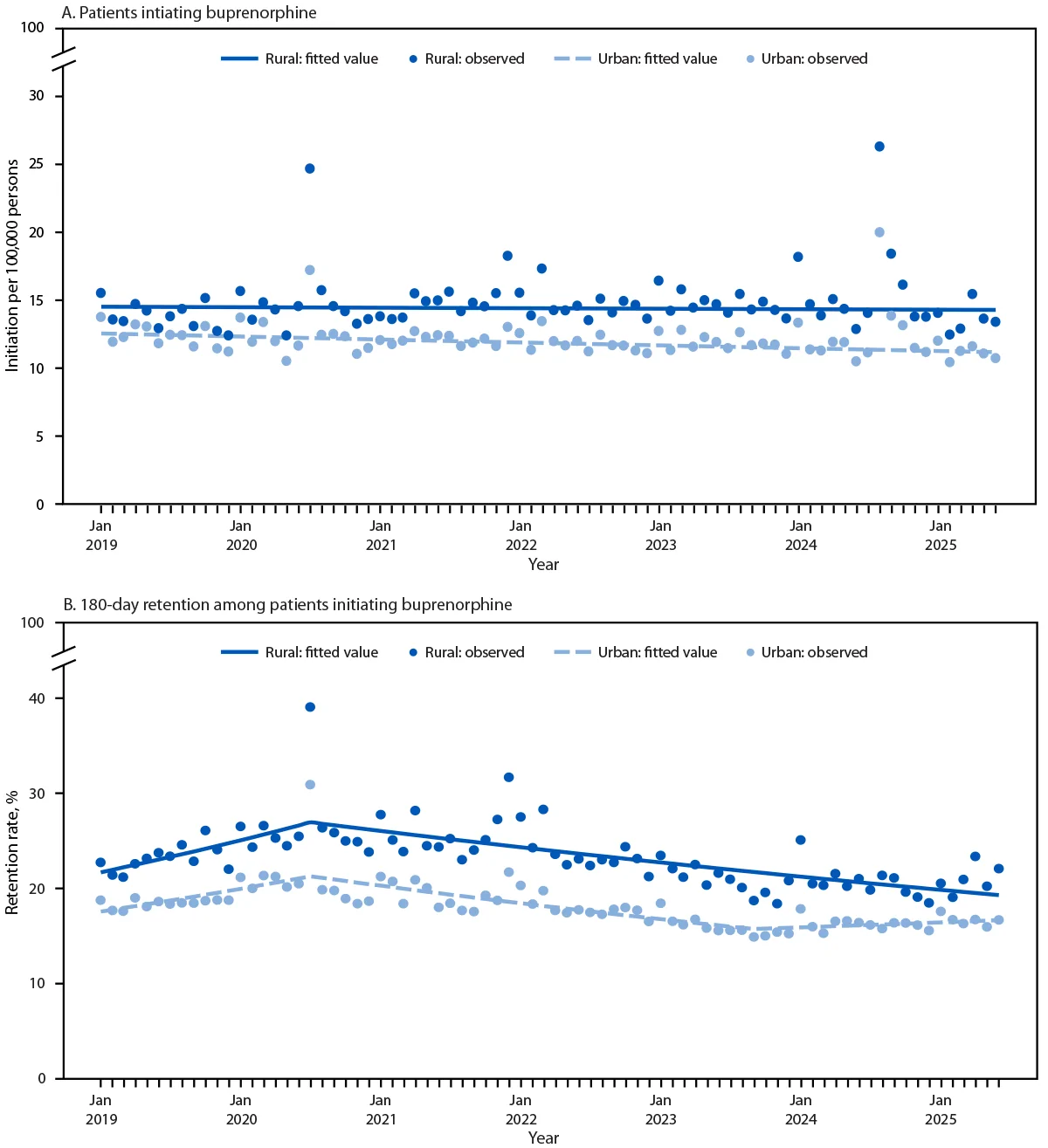
Buprenorphine initiation* (A) and retention^†^ (B) in urban and rural settings, by month — United States, 2019–2025 **Source:** IQVIA Longitudinal Prescription Database, 2019–2025. * Receipt of a buprenorphine prescription by a patient who had not received a prescription during the preceding 180 days. Initiations during July–December 2025 were excluded to ensure 180 days of follow-up data for all patients. ^†^ Continuous 180-day supply, which could be achieved through one or more prescriptions and refills, without a gap of >7 days.

**Initiation and retention.** Median monthly treatment initiation and 180-day treatment retention rates were higher in rural counties (14.2 per 100,000 persons and 23.1%, respectively) than in urban counties (11.8 per 100,000 persons and 17.7%, respectively) (Figure 1). Initiation declined in urban counties (AMPC = −0.2%) but remained stable in rural counties (AMPC = −0.02%). Retention increased in both rural and urban counties through July 2020, then declined in urban counties through September 2023 (MPC = −0.8%) before stabilizing and continued to decline in rural counties through June 2025 (MPC = −0.6%).

**FIGURE 2:**
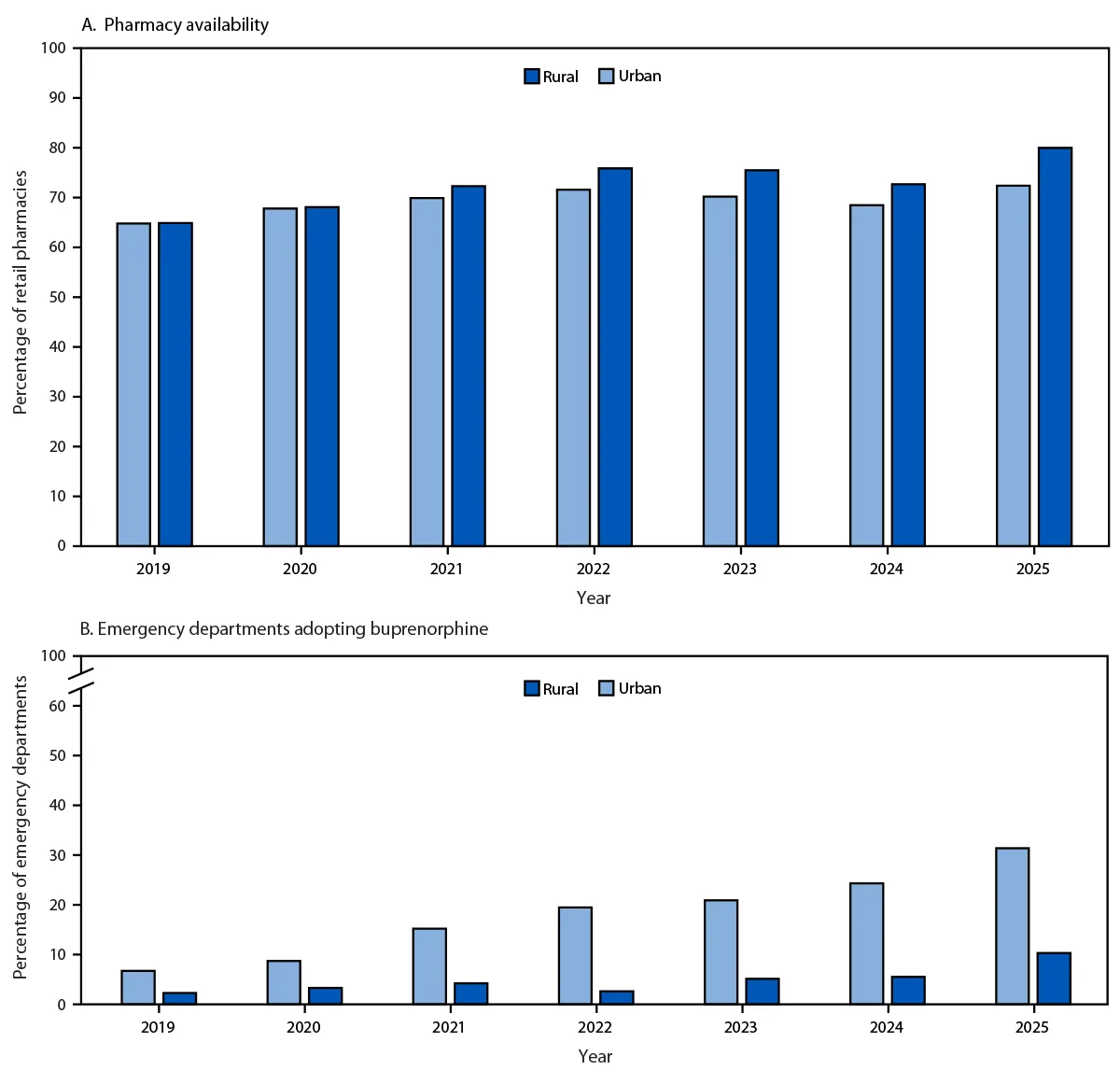
Retail pharmacy availability* of buprenorphine (A) and emergency departments adopting buprenorphine^†^ (B) in urban and rural settings, by year — United States, 2019–2025 **Source**: IQVIA Longitudinal Prescription Database and Premier Healthcare Database, 2019–2025. * The dispensing of at least one prescription for buprenorphine during each month a pharmacy was in operation in a given year. Percentages were calculated by dividing the number of pharmacies dispensing buprenorphine by the total number of pharmacies in operation during the same year. ^†^ Administration of buprenorphine to at least one person each month during which an opioid use disorder or overdose visit occurred during the year.

**Pharmacy availability of buprenorphine.** During 2019–2025, pharmacy availability of buprenorphine increased in urban counties (from 64.8% to 72.4%; AAPC = 1.4%). Availability also increased in rural counties (from 64.9% to 80.0%; AAPC = 2.7%) (Figure 2).

### Emergency Department Adoption and Administration

ED adoption increased from 6.7% to 31.4% in urban counties (AAPC = 30.3%) and from 2.3% to 10.3% in rural counties (AAPC = 23.3%) (Figure 2). Rates of ED-administered buprenorphine also increased in both urban counties (from 0.3 to 1.0 per 1,000 ED visits; AAPC = 18.4%) and rural counties (from 0.4 to 0.7; AAPC = 6.9%). Among ED visits for OUD or nonfatal opioid overdose, rates of buprenorphine ED administration increased from 33.9 to 111.1 in urban counties (AAPC = 23.3%) and from 25.2 to 103.3 in rural counties (AAPC = 25.1%) (Table).

## Discussion

Buprenorphine dispensed by retail pharmacies remained consistently higher in rural than in urban counties during 2019–2025, with larger increases in rural counties during 2019–2021. These patterns might reflect benefits of COVID-19–related telehealth flexibilities, particularly in areas with longer travel distances and more limited access to specialty care (*5*,*6*). Higher dispensing rates in rural areas might also indicate increased reliance on office-based buprenorphine, because opioid treatment programs are less available in rural areas (*5*). Together, these findings suggest that office-based prescribing is a particularly important treatment pathway in rural communities, where fewer specialty providers and more formidable geographic barriers might limit access to other forms of OUD care (*5*,*6*). Dispensing declined in both urban and rural counties after 2021, likely reflecting multiple factors such as changes in health care use and increasing days’ supply per prescription, whereas persistent barriers to access might have constrained the overall number of persons who received treatment (*3*).

Average days’ supply increased both in urban and rural counties, possibly reflecting evolving clinical practice and policy changes, including removal of previous authorization for medication for OUD in several states (*7*). Longer prescription durations might improve treatment continuity by reducing refill frequency, particularly in rural areas with more barriers to access (*5*). Higher retention in rural counties might reflect both more days’ supply and selection effects, because patients who successfully overcome more formidable access barriers might be more likely to remain engaged in care. Although treatment duration should be individualized, sustained engagement in buprenorphine treatment is associated with improved outcomes, underscoring the importance of ongoing patient-provider discussions to guide treatment duration and support continued engagement in treatment.

Average daily buprenorphine dose increased modestly, possibly reflecting increased recognition that some patients exposed to fentanyl, now the predominant opioid in the illegal drug supply, might benefit from higher buprenorphine doses (*8*). In 2024, FDA clarified that buprenorphine labeling does not specify a maximum dose and that dosing should be individualized based on patient need. Pharmacy availability of buprenorphine also increased in urban and rural counties and might be especially important in rural areas, where a higher reliance on pharmacies for the medication and limited access can constrain treatment across larger geographic areas.

The number of buprenorphine prescribers increased, whereas patients per prescriber and prescriptions per prescriber declined. This trend suggests broader distribution of prescribing capacity after elimination of the buprenorphine waiver requirement in December 2022 (*9*), although prescribing remained concentrated among high-volume prescribers. However, lower prescriber density in rural areas highlights persistent workforce shortages and the need to strengthen rural workforce capacity.

ED-administered buprenorphine increased in both urban and rural counties, likely reflecting growing awareness, supportive policies, and implementation strategies such as provider education, peer support, and clinical decision support tools (*10*). Despite these gains, adoption remained low, particularly in rural counties, where only one in 10 EDs in the sample had adopted buprenorphine in 2025. Low adoption suggests missed opportunities for treatment and persistent implementation barriers, such as limited behavioral health support, care coordination infrastructure, and staffing needed to implement protocols for OUD medication administration (*6*,*10*). ED-based administration can strengthen linkage to care by connecting patients directly to follow-up treatment before discharge, providing support from trained peer recovery specialists with lived experience, and offering case management to help coordinate ongoing services (*10*).

### Limitations

The findings in this report are subject to at least six limitations. First, prescriptions dispensed outside retail pharmacies (e.g., mail-order or clinic-based pharmacies) were not included in this analysis, which might have resulted in an underestimation of buprenorphine prescribing. Second, analyses were based on prescriber or ED location rather than patient residence, which could have resulted in misclassification of urban-rural treatment patterns if patients traveled across county boundaries to receive care. Third, Premier Healthcare Database hospitals represent a large, geographically diverse sample but are not nationally representative; therefore, findings might not be generalizable to all U.S. EDs or fully reflect urban-rural differences. Fourth, the analysis was limited to buprenorphine for OUD and did not include methadone or extended-release naltrexone; therefore, the findings do not represent all medications for OUD access or prescribing practices. Fifth, pharmacy availability was based on consistent dispensing of buprenorphine as a proxy because pharmacy inventory data were not available; therefore, this measure might not represent real-time availability. Finally, the absence of prescribing indications limited insight into clinician decision-making.

### Implications for Public Health Practice

Urban and rural counties showed distinct patterns of buprenorphine access. Pharmacy dispensing, initiation, and retention were higher in rural counties; however, the number of prescribers relative to the population and rates of ED-administered buprenorphine were higher in urban counties. These findings highlight opportunities to expand use of a low threshold for buprenorphine prescriptions, strengthen pharmacy availability, and, particularly in rural areas, support ED-based treatment administration and sustain telehealth flexibilities.

## Footnotes

* IQVIA data sources include IQVIA Xponent, IQVIA Longitudinal Prescriptions, and IQVIA Prescriber Level Xponent.

† Pharmacy availability of buprenorphine was operationally defined using dispensing as a proxy. Percentages were calculated by dividing the number of pharmacies dispensing buprenorphine by the total number of pharmacies in operation during the same year.

§ The U.S. Census Bureau defines an urban area as a territory in which core census groups or blocks have a population density of at least 1,000 persons per square mile and surrounding census blocks have an overall density of at least 500 persons per square mile. Rural areas are considered territory outside the definition of urban.

¶ 45 C.F.R. part 46.102(l)(2), 21 C.F.R. part 56; 42 U.S.C. Sect. 241(d); 5 U.S.C. Sect. 552a; 44 U.S.C. Sect. 3501 et seq.

## References

[1] Krawczyk N, Mojtabai R, Stuart EA, Opioid agonist treatment and fatal overdose risk in a state-wide US population receiving opioid use disorder services. Addiction 2020;115:1683–94. 10.1111/add.14991 32096302 PMC7426244

[2] Dowell D, Brown S, Gyawali S, Treatment for opioid use disorder: population estimates—United States, 2022. MMWR Morb Mortal Wkly Rep 2024;73:567–74. 10.15585/mmwr.mm7325a1 38935567 PMC11254342

[3] Guy GP Jr, Jones CM, Rikard M, Strahan AE, Zhang K, Olsen Y. Individuals dispensed buprenorphine in the US before and after federal policy changes aimed at increasing access. J Addict Med 2025;19:615–20. 10.1097/adm.0000000000001457 39969964 PMC12353755

[4] Ramdin C, McGowan T, Perrone J, Mazer-Amirshahi M, Nelson LS. Buprenorphine administration and prescribing at emergency departments: a national analysis from 2014–2021. J Addict Med 2025;19:254–9. 10.1097/adm.0000000000001402 39514888

[5] Amiri S, Hirchak K, McDonell M, Denney J, Buchwald D, Amram O. Access to medication-assisted treatment in the United States: comparison of travel time to opioid treatment programs and office-based buprenorphine treatment. Drug Alcohol Depend 2021;1:224–26.https://doi.org/10.1016/j.drugalcdep.2021.108727 10.1016/j.drugalcdep.2021.108727 33962300

[6] Morenz AM, Nance RM, Mixson LS, Barriers to accessing medications for opioid use disorder among rural individuals. Int J Drug Policy 2025;140:104805. 10.1016/j.drugpo.2025.104805 40252371 PMC12103983

[7] Hu JC, Hutchings K, Jalali A, Kapadia S, Bao Y, Underhill K. State laws prohibiting prior authorization for medications for opioid use disorder in private insurance, 2015–2023. Health Aff (Millwood) 2025;44:1369–77. 10.1377/hlthaff.2025.00191 41183241 PMC12721913

[8] Snyder H, Chau B, Kalmin MM, High-dose buprenorphine initiation in the emergency department among patients using fentanyl and other opioids. JAMA Netw Open 2023;6:e231572. 10.1001/jamanetworkopen.2023.1572 36867410 PMC9984967

[9] Guy GP Jr, Jones CM, Rikard SM, Zhang K, Olsen Y. Prescriber-level changes in buprenorphine dispensing in the USA before and after federal policy changes aimed at increasing prescribing. J Gen Intern Med 2025;40:4090–3. 10.1007/s11606-025-09655-8 40562887 PMC12686323

[10] Hawk K, Hoppe J, Ketcham E, Consensus recommendations on the treatment of opioid use disorder in the emergency department. Ann Emerg Med 2021;78:434–42. 10.1016/j.annemergmed.2021.04.023 34172303

